# Diffusion-Weighted MRI Reflects Proliferative Activity in Primary CNS Lymphoma

**DOI:** 10.1371/journal.pone.0161386

**Published:** 2016-08-29

**Authors:** Stefan Schob, Jonas Meyer, Matthias Gawlitza, Clara Frydrychowicz, Wolf Müller, Matthias Preuss, Lionel Bure, Ulf Quäschling, Karl-Titus Hoffmann, Alexey Surov

**Affiliations:** 1 Department of Neuroradiology, University Hospital Leipzig, Leipzig, Germany; 2 Department of Neuropathology, University Hospital Leipzig, Leipzig, Germany; 3 Department of Diagnostic and Interventional Radiology, University Hospital Leipzig, Leipzig, Germany; 4 Department of Radiology, Martin Luther University of Halle-Wittenberg, Halle-Wittenberg, Germany; 5 Department of Neurosurgery, University Hospital Leipzig, Leipzig, Germany; 6 Department of Radiology, McGill University Health Center, Montreal General Hospital, Montreal, Canada; University of Glasgow, UNITED KINGDOM

## Abstract

**Purpose:**

To investigate if apparent diffusion coefficient (ADC) values within primary central nervous system lymphoma correlate with cellularity and proliferative activity in corresponding histological samples.

**Materials and Methods:**

Echo-planar diffusion-weighted magnetic resonance images obtained from 21 patients with primary central nervous system lymphoma were reviewed retrospectively. Regions of interest were drawn on ADC maps corresponding to the contrast enhancing parts of the tumors. Biopsies from all 21 patients were histologically analyzed. Nuclei count, total nuclei area and average nuclei area were measured. The proliferation index was estimated as Ki-67 positive nuclei divided by total number of nuclei. Correlations of ADC values and histopathologic parameters were determined statistically.

**Results:**

Ki-67 staining revealed a statistically significant correlation with ADCmin (r = -0.454, p = 0.038), ADCmean (r = -0.546, p = 0.010) and ADCmax (r = -0.515, p = 0.017). Furthermore, ADCmean correlated in a statistically significant manner with total nucleic area (r = -0.500, p = 0.021).

**Conclusion:**

Low ADCmin, ADCmean and ADCmax values reflect a high proliferative activity of primary cental nervous system lymphoma. Low ADCmean values—in concordance with several previously published studies—indicate an increased cellularity within the tumor.

## Introduction

Primary lymphomas of the central nervous system account for approximately 4% of newly diagnosed brain tumors[1]. Representing a subgroup of Non-Hodgkin Lymphoma (NHL), more than 90% of them are classified as Diffuse Large B-Cell Lymphoma (DLBCL). Occurrence of primary CNS lymphoma (PCNSL) strongly correlates with immunosuppression, either iatrogenic, infectious or congenital[2]. Although PCNSL in immunocompromised subjects show an association with Epstein-Barr Virus (EBV), the incidence rate of PCNSL in immunocompetent subjects without EBV has increased sharply over the last thirty years[3]. PCNSL rarely extends beyond the CNS. This is postulated to be due to the immune privilege of the CNS[4]. The neoplastic lymphocytes are identified and removed from the immunologically more active periphery, but persist in the CNS where the immunological surveillance is comparatively less fine-meshed, enabling them to maintain active proliferation.

In clinical practice, Ki-67 is a widely used cellular marker for proliferative activity in lymphoma and other malignancies[5]. The nonhistone nuclear protein is synthesized throughout the whole cell cycle except the G0 phase, and has been shown to be responsible for cell division[6]. The prognostic value of Ki-67 in different histopathologic subtypes of lymphoma has been examined in multiple studies[5]. For example, a high level of Ki-67 expression is associated with a decreased overall survival but a good therapeutical response of subjects suffering from DLBCL following Rituximab administration. Sustained proliferation and enabled replicative immortality, both being general hallmarks of cancer[7] result in a high cellular density of the malignancy, which is a consistent finding in PCNSL[8].

Diffusion-Weighted Imaging (DWI), a technique based on phase-defocusing and phase refocusing gradients has been used to measure the motion of water molecules on the microspcopic scale. Besides its importance in stroke imaging, DWI plays a growing role in the characterization of brain tumors. Tumors with densely packed cells like astrocytoma, lymphoma and medulloblastoma[9] reveal an increased DWI signal on high b-values images and low ADC values due to the relative restriction of water caused by high cellularity in combination with high nuclear-to-cytoplasmic (N/C) ratios. The relation between low ADC values, high cellularity and poor outcome has already been shown for PCNSL[10]. However, not all studies in the field demonstrate an association between cellularity and ADC-values: Wu et al.[11] did not find a correlation between ADC values and cellularity in Diffuse Large B-Cell Lymphoma and Follicular Lymphoma. Although the relation between N/C ratio, cellularity and ADC values has been examined in PCNSL, the association between water diffusibility (ADCmin, ADCmax, ADCmean), proliferation rate (Ki-67 expression), total nuclear area, average nuclear area and cell count has not yet been elucidated.

The purpose of this study was i) to investigate if ADC values in PCNSL correlate with proliferative activity in terms of Ki-67 expression and ii) to further elucidate if ADC values (ADCmin, ADCmean and ADCmax) show correlations with nuclei count, total nucleic area or average nucleic size.

## Materials and Methods

### Patient Selection

All patients or caregivers gave their informed consent for the asservation of sample remnants and compiling of clinical and radiological data. Informed consent was given in writing. The study was approved by the local ethics committee (Ethikkommission Universität Leipzig, Az 330-13-18112013).

Potential patients in the period of January 2006 through March 2015 were identified on the basis of the diagnosis PCNSL conducting a full-text search in the database of the institutes for pathology and radiology, respectively. The search revealed 40 patients in the radiological database, all of which were biopsied in our hospital and had a consecutive histopathological workup. Only previously untreated patients with pretreatment DWI were included. None of the studied patients were immunodeficient. Due to a lack of comparability of DWI measurements between different MR scanners (Philips Intera, Philips Achieva and Siemens Symphony, all 1.5T) we excluded 19 patients from our analysis. 21 patients (9 female, 13 male; 28–89 years, mean age 68,5 years) met the inclusion criteria.

### MR Imaging

All images were obtained in the clinical routine workup by using a 1.5T MRI scanner (Siemens Magnetom Symphony 1,5T) with the standard Siemens head coil (CP head array, model #1P3146037). DWI was performed using a single-shot spin-echo (SE) echo planar sequence with following parameters: Echo time(TE)/Repetition time(TR) = 6000/105 ms, 90° flip angle, 57 transverse sections, slice thickness = 5 mm, field of view (FOV) = 230 mm. Diffusion-sensitizing gradients were applied sequentially in the x, y and z directions with b factors of 0 and 1000 s/mm^2^. ADCs were automatically calculated at the operating console of the MR scanner and displayed as corresponding ADC maps. Postcontrast T1-weighted 3D-gradient echo sequence(GRE) imaging was obtained with following parameters: TR/TE = 2150/3.93 ms, flip angle 15°, 1-mm section thickness and 230 mm FOV. A standard dose (0.1 mmol/kg body weight) of gadoteric acid (Gd-DOTA, Dotarem; Laboratoire Guerbet, Aulnay-sous-Bois, France) was injected intravenously. Routine anatomic precontrast T1/ T2_tirm_tra_dark_fluid (TR/TE = 9000/114, slice thickness 5mm, flip angle 150°, 28 transverse sections) images were also obtained.

### MR Image Analsysis

All images were available in digital format. Image analysis was performed on a Siemens SIENET MagicView 1000 console previous to stereotactic biopsy. The lesion in question was determined by the stereotactic neurosurgeon. After stereotactic biopsy postinterventional images were compared with preoperative images to confirm that the analysed lesion corresponded to the biopsied portion of the tumor. B0, B1000 and ADC maps were coregistered with the postcontrast T1-weighted 3D-gradient echo sequence to improve visualization and correlation. Subsequently the readers reviewed the coregistered B0, B1000 and ADC maps and drew a single circular region of interest (ROI)within the enhancing part of the tumor corresponding to the biopsied portion. ROI size was determined depending on the size of the post-biopsy lesion, the lower limit being 0.2cm^2^, the upper limit 0.49cm^2^. Hemorrhage, cysts and necrosis were avoided when drawing the ROIs. ADC min, max and mean were measured on the corresponding maps. Additionally, deltaADC (deltaADC = ADCmax—ADCmin) was calculated for each tumor. The used technique for measuring ADC values was chosen because of its robustness and clinical feasibility.

### Histopathological Analysis

In every case the diagnosis of cerebral lymphoma was confirmed by surgical biopsy.

Ki-67 antigen stained specimens (MIB-1 monoclonal antibody, DakoCytomation, Denmark) were re-analyzed in this study. All images were digitalized by using a research microscope Jenalumar with camera Diagnostic instruments 4.2 (Jena, Germany). The tumor proliferation index was estimated as following relation: number of specifically stained nuclei divided by all nuclei. The area showing the highest number of positive cell nuclei was selected in each case.

Cell count was calculated as total number of nuclei per two high power fields (x400).

In addition, the histological samples were analyzed for estimation of total and average nucleic areas by using ImageJ software 1.48v (NIHS) as described in previous reports [12]. Tumor cell nuclei were easily identified by their color intensities. Total and average nucleic areas were automatically calculated in two high power fields (x400).

### Statistical analysis

Statistical analysis and graphics creation were performed using SPSS version 22. Collected data was evaluated by means of descriptive statistics. Spearman-Rho correlation coefficient was used to analyze the association between ADC values and histological parameters. P-values < 0.05 were taken to indicate statistical significance.

## Results

### DWI measurements and histopathological analysis

All investigated PCNSL were located supratentorially and showed close proximity to the lateral ventricles. The size of the lesions ranged from 1.2cm to 7.8cm. 6 out of 21 patients had multifocal supratentorial lesions. As described above only the stereotactically biopsied part of the tumor was analysed with MRI.

**Fig 1:**
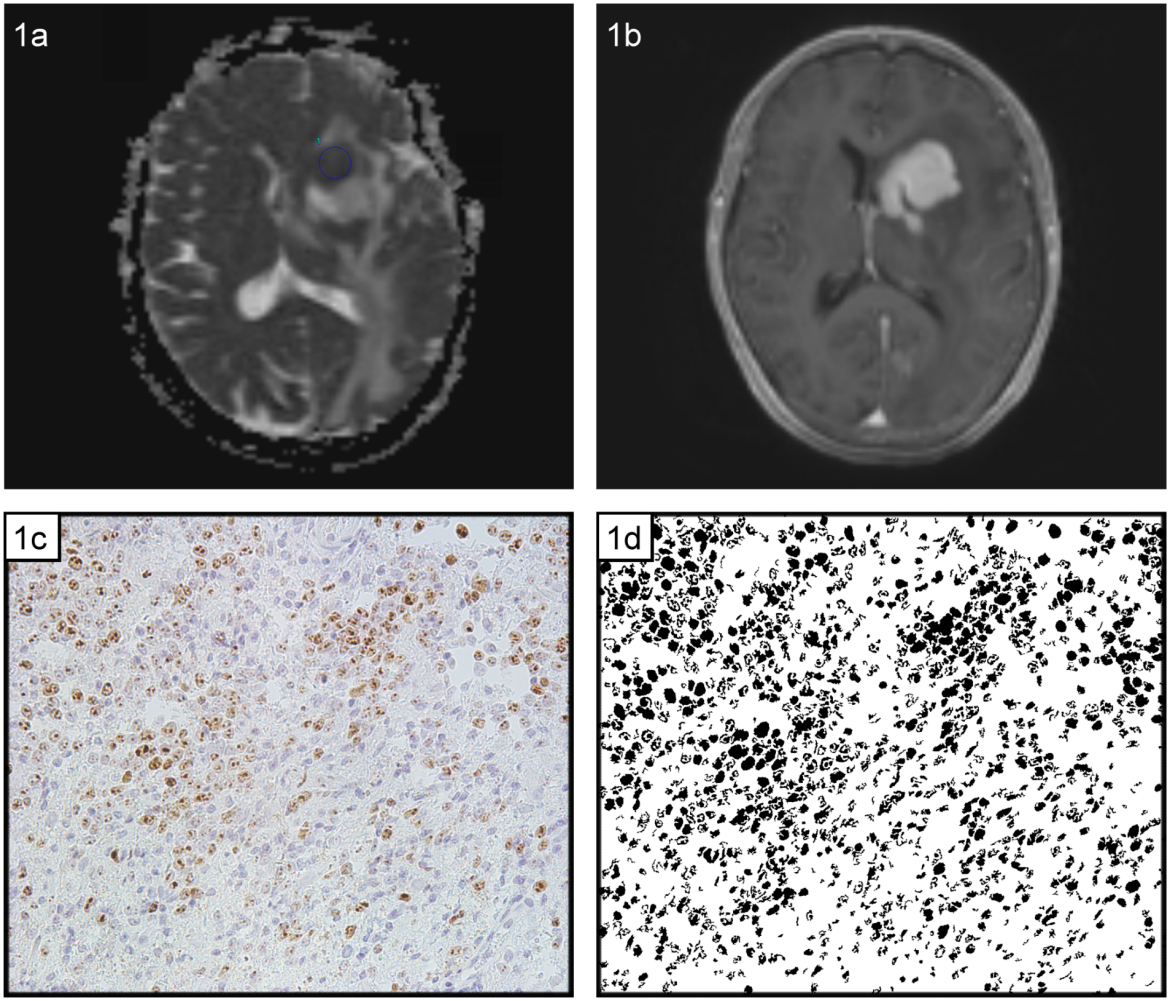
MRI and histopathological findings in a patient with diagnosed cerebral lymphoma. a. T1w image documenting a lesion with marked homogenous enhancement. b. ADC map. The ADCmin value of the lesion is 64x10^-4^mm^2^s^-1,^ the ADCmean value of the lesion is 80×10^−4^ mm^2^s^-1^ and the ADCmax value is 133×10^−4^ mm^2^s^-1^. c. Immunohistochemical stain (MIB-1 monoclonal antibody). Ki 67-index = 50%. d. “Particles tool” analysis image (ImageJ converted MIB-1 image). Cell count (total number of nuclei in the image), total nucleic area (total area of all nuclei in the image) and average nucleic area (total area of all nuclei in the image divided by the number of nuclei in the image) were calculated via the particles tool in ImageJ. For the image shown in fig 1d the following values were determined by the “Particles tool” analysis; cell count = 1424, total nucleic area = 105678.14 μm^2^ and average nucleic area = 74.37 μm^2^.

Fig 1a exemplarily shows a representative T1 weighted spin echo image of a CNS lymphoma after gadolinium administration. Fig 1b shows the corresponding ADC map.

Table 1 displays gender, age and ADC fractions. Table 2 summarizes ADC values, cell count, Ki-67 expression, total nucei area and average nuclei area of all PCNSL patients.

**Table 1:** Summary of age, sex and ADC values (ADCmin, ADCmean, ADCmax and deltaADC) of each lesion corresponding to the respective individuum measured as described above. ADCmin, ADCmean, ADCmax and deltaADCvalues displayed as × 10^−4^ mm^2^s^-1^.

Sex	Age (years)	ADCmin (10^−4^ mm^2^s^-1^)	ADCmax (10^−4^ mm^2^s^-1^)	ADCmean (10^−4^ mm^2^s^-1^)	deltaADC (10^−4^ mm^2^s^-1^)
F	64	64	152	87	51
M	73	53	158	98	108
M	78	72	123	88	127
M	59	65	174	115	50
F	74	85	224	150	82
F	28	51	123	71	69
F	89	64	133	80	68
F	80	60	110	85	109
M	79	64	133	87	73
M	86	58	126	73	67
M	67	70	141	91	71
M	64	77	190	140	88
M	55	49	170	74	121
M	82	66	139	87	72
F	77	77	157	107	69
M	73	54	121	87	80
M	32	65	147	89	139
M	64	35	120	80	85
F	78	70	178	118	113
F	55	60	121	81	105
M	65	63	190	114	61

**Table 2:** Synopsis of ADC Fractions and histopathological parameters.

	N	Minimum	Maximum	Average Value	Standard Deviation
**ADCmin** (10^−4^ mm^2^s^-1^)	21	35.00	85.00	62.95	11.01
**ADCmax** (10^−4^ mm^2^s^-1^)	21	107.00	224.00	146.67	30.92
**ADCmean** (10^−4^ mm^2^s^-1^)	21	71.31	157.00	97.83	25.12
**deltaADC** (10^−4^ mm^2^s^-1^)	21	50.00	139.00	86.10	25.37
**Nuclei count**	21	319.00	1922.00	1288.62	366.69
**Total nuclei area** (μm^2^)	21	19988.01	216517.76	106617.71	44549.13
**Average nuclei area** (μm^2^)	21	53.20	267.91	86.52	46.41

In brief, the ranges of ADC values were as follows: ADCmin = 35.0–85.0 10^−4^ mm^2^s^-1^ (mean value 62.95x10^-4^ mm^2^s^-1^), ADCmean = 71.31–157.00x10^-4^ mm^2^s^-1^ (mean value 97.82x10^-4^ mm^2^s^-1^), ADC max = 107.00–224.00x10^-4^ mm^2^s^-1^ (mean value 147.66x10^-4^ mm^2^s^-1^) and deltaADC = 50.00–139.00x10^-4^ mm^2^s^-1^ (mean value 86.10x10^-4^ mm^2^s^-1^). Most lesions exhibited high proliferation rates ranging from 50% to 95% with a mean value of 76.19% Ki67 expressing nuclei. Fig 1c exemplarily shows Ki-67 staining of a primary CNS lymphoma. The immunostained section corresponds to the lymphoma shown in fig 1a and 1b.

Total nuclei area was ranging widely from 19988.01 to 216517.76 μm^2^ with a mean value of 106617.71 μm^2^.

Interestingly, average nuclei areas had a large range as well, varying from 53.20 to 267.91μm^2^ with a mean value of 86.52 μm^2^.

Fig 1d exemplarily shows the particles tool analysis image corresponding to the lymphoma shown in fig 1a, 1b and 1c.

### Correlation analysis

**Fig 2:**
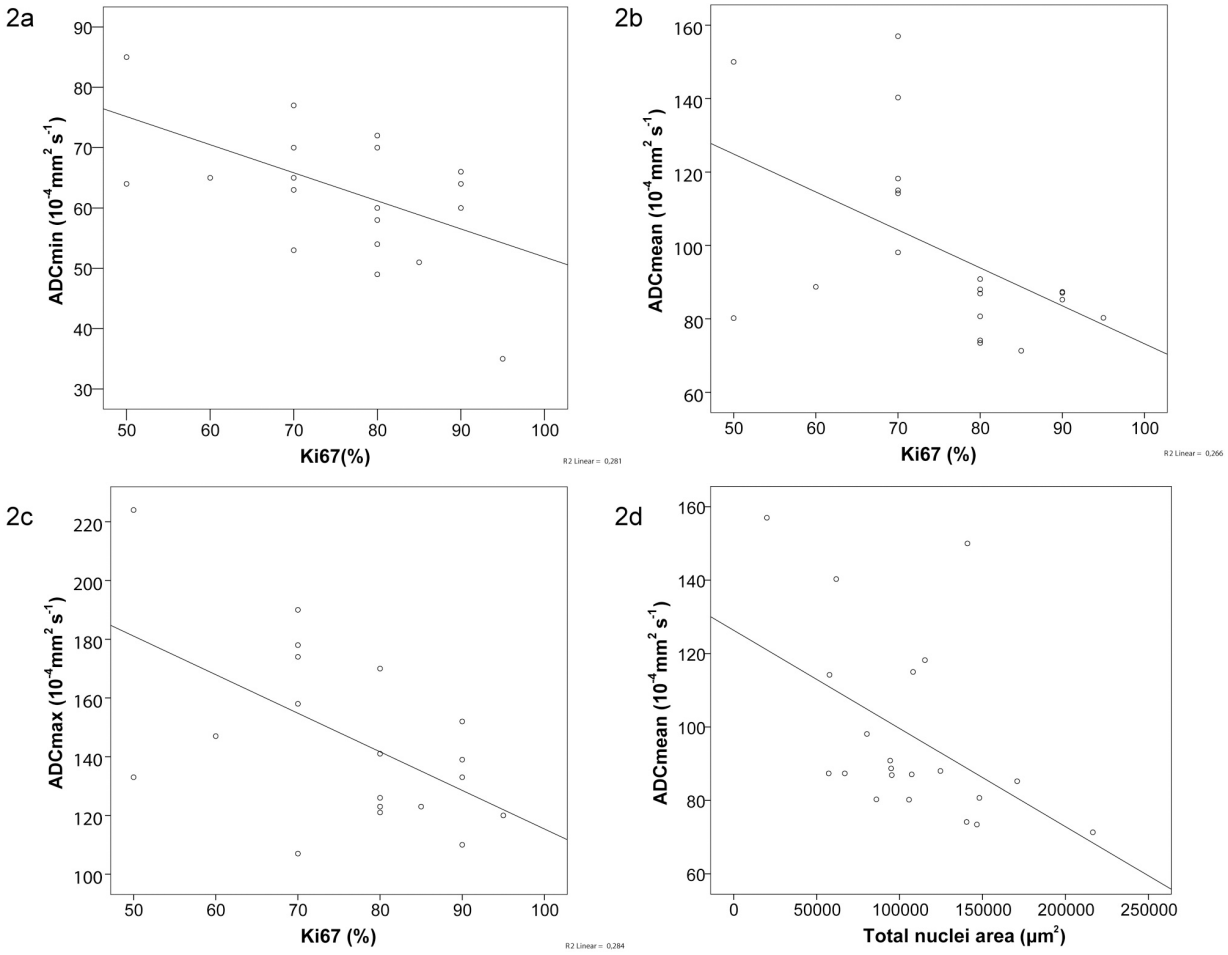
Significant correlations between ADC fractions (displayed as 10^−4^ mm^2^s^-1)^ and histopathological parameters (Ki-67: positively stained nuclei displayed in % and total nuclei area: displayed in μm^2^). Statistical analysis documenting significant correlations between ADCmin and Ki-67 level (r = -0.454; p = 0.038) (a), ADCmean and Ki-67 level (r = -0.546; p = 0.010) (b), ADCmax and Ki-67 level (r = -0.515; p = 0.017). Significant correlations between ADCmean and total nuclei area (r = -0.500; p = 0.021) (d) are also shown.

Table 3 summarizes the statistical analysis of the relation between histopathological parameters and ADC fractions, displaying the following findings: i) ADCmin showed a statistically significant correlation with Ki-67 expression (r = -0.454, p = 0.038). No correlations were found for ADCmin with nuclei count (r = -0.127, p = 0.584), total nuclei area (r = -0.228, p = 0.312) or average nuclei size (r = -0.173, p = 0.455). ii) ADCmean showed statistically significant correlations with Ki-67 expression (r = -0.546, p = 0.010) and total nuclei area (r = -0.500, p = 0.021). No correlations were found between ADCmean and nuclei count (r = -0.340, p = 0.132) or average nuclei size (r = -0.289, p = 0.204). iii) ADCmax showed a statistically significant correlation with Ki-67 expression (r = -0.515, p = 0.017). No correlations were found for ADCmax with nuclei count (r = -0.012, p = 0.960), total nuclei area (r = -0.155, p = 0.501) or average nuclei area (r = -0.144, p = 0.534). DeltaADC showed no statistically significant correlations with the investigated histopathological features (deltaADC and nuclei count: r = -0.223, p = 0.332; deltaADC and total nuclei area: r = -0.310, p = 0.172; deltaADC and average nuclei area: r = -0.212, p = 0.357), although a trend was delineable for deltaADC and Ki-67 (r = -0.428, p = 0.053). Fig 2 displays scatterplots graphically demonstrating the statistically significant relationships between DWI fractions and histopathological parameters.

**Table 3:** Identified correlations between diffusion and histopathological parameters, statistically significant findings (p<0.05) are displayed in bold italics.

Parameter	Cell count		Ki-67 (%)		Total Nuclei Area (μm^2^)		Average Nuclei Area *μm^2^)	
	r =	p =	r =	p =	r =	p =	r =	p =
**ADCmin** (10^-4^mm^2^s^-1^)	-0.127	0.584	***-0*.*454***	***0*.*038***	-0.228	0.321	-0.173	0.455
**ADCmean** (10^-4^mm^2^s^-1^)	-0.340	0.132	***- 0*.*546***	***0*.*010***	***-0*.*500***	***0*.*021***	-0.289	0.204
**ADCmax** (10^-4^mm^2^s^-1^)	0.012	0.960	***-0*.*515***	***0*.*017***	-0.155	0.501	-.0144	0.534
**deltaADC** (10^4^mm^2^s^-1^)	-0.223	0.332	-0.428	0.053	-0.310	0.172	-0.212	0.357

## Discussion

To the best of our knowledge the inverse correlation between different ADC fractions and proliferative activity of PCNSL has not been elucidated yet.

The present study identified significant correlations between ADCmin, ADCmean and ADCmax measurements and cellular Ki-67 expression in biopsied portions of PCNSL, thus reflecting tumor biology in terms of proliferative activity. Furthermore, the study validated in vivo measured, low ADCmean values as a surrogate for tumor portions with increased nuclei area, reflecting increased cellularity.

The significance of DWI for evaluation of PCNSL and differentiation from other brain lesions has grown considerably over the years. An early study by Cotton and colleagues investigated 9 biopsy-proven cases of cerebral lymphoma and found that the lesions were hyperintense in DWI with normal or decreased ADC values[13]. A later study by Zacharia and coworkers found that restricted diffusion with decreased ADC values is a consistent finding of PCNSL in immunocompetent patients prior treatment[8]. Haldorsen et al. discussed that DWI provides a potentially valuable tool to distinguish between glioma, metastases and PCNSL based on cellularity-related diffusion restriction[14]. Kickingereder and colleagues found that all ADC fractions (min, max and mean) were lower in PCNSL compared to glioblastoma[15]. Mabray and coworkers demonstrated that the combined use of ADC and CSF biomarkers (CXC chemokine ligand 13 and interleukin 10) increased the diagnostic performance for the diagnosis of PCNSL [16].

Although diffusion restriction is a consistent imaging finding in lymphoma, the question whether in vivo measured ADC values reflect cellularity in lymphoma has been answered differently in previous studies. For example, Wu and colleagues[9,11,17] showed that follicular lymphoma and diffuse large B-cell lymphoma did not exhibit statistically different ADC values, whereas the corresponding histopathological investigation revealed that the cellularity of both entities varied greatly in a statistically significant manner. Contrary to this, Guo et al.[9] showed that cellularity and ADC values correlated inversely in a statistically significant manner after investigation of high grade astrocytomas and PCNSL.

The results of our study concur with the findings of Guo and colleagues, validating the link between decreased water diffusibility in lymphomas measurable in MRI based on high cellularity in histology. Values of ADC fractions in our study are approximately ten times larger than those given by Guo et al.[9] and Cotton et al.[13]. We attribute this difference to the fact that different MRI scanners and coils were used in these studies. Guo and colleagues used a 1.5T GE platform, Cotton and colleagues used a 1.5T Philips MRI scanner and we used a 1.5T Siemens scanner. As shown by Sasaki et al.[18] and Kivrak et al.[19], absolute ADC values can substantially vary among different coil systems, imagers, vendors, and magnetic field strengths.

Although numerous studies provide evidence for the relationship of low ADC values and high cellularity, the question which ADC parameter—ADCmin, ADCmean or ADCmax best reflects the actual cellular density in a malignant tumor has not been answered conclusively. Chen and coworkers[17,20] conducted a meta analysis investigating the relationship between DWI and cellularity in different types of malignant tumors and found a strong, inverse correlation between ADC and cellularity especially in brain tumors including PCNSL. According to Chen et al. an overall of 14 studies examined the correlation of cellularity and restricted water diffusibility in brain tumors. Three of these studies showed that ADCmin only reflected increased cellularity. Ten out of fourteen studies showed that ADCmean only reflected increased cellularity. Only one study showed that both, ADCmin and ADCmean indicated high tumor cellularity. The fact that in few studies ADCmin alone reflected high cellularity, whereas in the majority of the studies ADCmean (but not ADCmin) indicated high cellularity raises the question as to how this discrepancy can be explained. Some studies did not reveal the exact method for ADC measurement, generally ADC values were measured based on ROIs corresponding to the whole contrast agent enhancing part of the malignancy. These values were correlated to the histologically derived data (e.g. cell count, nucleus-cytoplasm-ratio), which represented only the very small, biopsied portion of the tumor. Since most of the malignant tumors are composed of more aggressively infiltrating, rapidly dividing portions exhibiting high cellularity and less biologically active portions, potentially hemorrhage and necrosis as well, it is of great importance to only correlate the local apparent diffusion coefficient that corresponds to the biopsied, histologically examined part of the malignancy. As a consequence of the aforementioned we assume that the varying correlations being found between ADCmin and cellularity or ADCmean and cellularity, respectively, express differences in tumor heterogeneity between the different studies. Since we assume that ROIs, which extend to the whole contrast agent enhancing part of the tumor (including significantly more than the histologically investigated part) may result in rather unspecific ADC measurements, we identified the biopsied part of each PCNSL, measured the corresponding ADCmin, ADCmean and ADCmax values and correlated them with nuclei count, total nuclei area and average nuclei area. We found a conclusive, statistically significant correlation of ADCmean and total nuclei area, which concurs with the majority of the studies investigated by Chen and colleagues, indicating ADCmean as a good in vivo measurable surrogate for increased cellularity.

In the second step this study revealed that ADCmin, ADCmean and ADCmax exhibit statistically significant inverse correlations with Ki-67 expression, thus providing evidence of low ADC values as in vivo surrogates for increased proliferative activity of PCNSL. This is in accordance with the work of Guzmán-De-Villoria et colleagues[21], who found a trend of correlation between low ADCmean values and Ki-67expression in glioma and other brain tumors. We assume that the relationship between ADC values and Ki-67 expression in gliomas is more difficult to prove, since the tissue architecture of glioma is more heterogeneous and the resulting ADC values exhibit a greater degree of variation compared to PCNSL. Other investigators were able to determine that increasing ADC values indicate positive therapeutic responses of tumors to antiproliferative treatment[22]. Further studies were able to show that low ADC values were good indicators for the efficacy of specific, proliferation-inhibitory drugs[23,24]. With reference to our findings we hypothesize that the antiproliferative effect of chemotherapeutic drugs results in a decrease of Ki-67 positive, proliferating cells and finally in a reduction of overall tumor cellularity—thus leading to a measurable increase of water diffusibility in MRI. This assumption is based on the results of Ho et al.[25] who were able to demonstrate that tumor areas with low ADC values exhibit increased glucose-uptake, thus being more metabolically active.

As reported by Surov et al., different histopathological features are associated with different DWI parameters in meningioma[12]. Based on these findings and the results of our present study we hypothesize that—due to the comparatively homogeneous cellular architecture of CNS lymphoma—all ADC parameters in CNS lymphoma are influenced by the proliferative activity of the tumor. In a recently published study we were able to show that expression of the water channel Aquaporin 4 in meningioma correlates well with ADCmax values[26] We thereupon assume that in different entities (lymphoma, meningioma, glioma etc.), diverse histopathological and cyto-architectural features influence ADC parameters to a varying degree.

Our study had several limitations. The main limitation of our study is the use of perfusion-sensitive ADC values. In the standardized clinical setting, DWI of the brain is conventionally performed with a low (b = 0mm^2^/s) and a high b-value (b = 1000mm^2^/s). Diffusion-weighted data acquired over a range of b values that includes low b values (< 100–150 s/mm^2^) are sensitive to signal attenuation from capillary perfusion[27]. Perfusion-insensitive diffusion weighted data can be obtained by using only higher b-values (>150 mm^2^/s)[27]. As shown by Priola et al., the use of perfusion-insensitive ADC measurements significantly improves diagnostic accuracy of DW-MRI[28]. Based on the fact that contrast enhancement was used as reference for ROI positioning, the ADC values obtained in our study may be overestimated due to perfusion effects[29,30]. Furthermore, the results of our study were possibly influenced by the small sample size and the retrospective design. Additionally, only a small portion of the contrast enhancing part of the mass was biopsied and investigated histopathologically, which does not reflect probable tumor heterogeneity. The applied technique for measuring ADC values was chosen because of its robustness and clinical feasibility.

## Conclusion

This is the first study to show that ADCmin, ADCmean and ADCmax values are associated with high proliferation rates of PCNSL, which indicates a promising potential for ADC measurements as in vivo markers for treatment response or disease relapse. Furthermore, this study confirmed ADCmean as best in vivo surrogate of DWI for cellular density in PCNSL.
